# Unravelling the Chemical Influence of Water on the PMMA/Aluminum Oxide Hybrid Interface *In Situ*

**DOI:** 10.1038/s41598-017-13549-z

**Published:** 2017-10-17

**Authors:** Sven Pletincx, Kristof Marcoen, Lena Trotochaud, Laura-Lynn Fockaert, Johannes M. C. Mol, Ashley R. Head, Osman Karslioğlu, Hendrik Bluhm, Herman Terryn, Tom Hauffman

**Affiliations:** 1 Department of Electrochemical and Surface Engineering (SURF), Vrije Universiteit Brussel, Pleinlaan 2, 1050 Brussels, Belgium; 2 Chemical Sciences Division, Lawrence Berkeley National Laboratory, One Cyclotron Road, Berkeley, CA 94720 United States of America; 3 Department of Materials Science and Engineering, Delft University of Technology, Mekelweg 2, 2628 CD Delft, The Netherlands

## Abstract

Understanding the stability of chemical interactions at the polymer/metal oxide interface under humid conditions is vital to understand the long-term durability of hybrid systems. Therefore, the interface of ultrathin PMMA films on native aluminum oxide, deposited by reactive adsorption, was studied. The characterization of the interface of the coated substrates was performed using ambient pressure X-ray photoelectron spectroscopy (APXPS), Fourier transform infrared spectroscopy in the Kretschmann geometry (ATR-FTIR Kretschmann) and time-of-flight secondary ion mass spectrometry (ToF-SIMS). The formation of hydrogen bonds and carboxylate ionic bonds at the interface are observed. The formed ionic bond is stable up to 5 Torr water vapour pressure as shown by APXPS. However, when the coated samples are exposed to an excess of aqueous electrolyte, an increase in the amount of carboxylate bonds at the interface, as a result of hydrolysis of the methoxy group, is observed by ATR-FTIR Kretschmann. These observations, supported by ToF-SIMS spectra, lead to the proposal of an adsorption mechanism of PMMA on aluminum oxide, which shows the formation of methanol at the interface and the effect of water molecules on the different interfacial interactions.

## Introduction

One of the main goals in interface engineering is the achievement of high adhesion strengths at polymer/metal interfaces, especially in humid or aggressive conditions^1^. Which factors of interfacial contributions actually result in a high macroscopic adhesion is the topic of an ongoing debate. Although mechanical interlocking has mainly been put forward as the main force holding hybrid structures, it becomes increasingly clear that interfacial chemical interactions of surface hydroxyl groups and functional groups of polymers are key factors in hybrid structure durability^2–9^.

At the interface of these two regions, intermolecular interactions result in chemical bond formation, which determine the performance of coatings or adhesives as they have to withstand high mechanical forces and corrosive attacks typically for years and even decades^10,11^. A good understanding of the local chemistry of these interactions is thus required.

To investigate the interface region in an adequate way, one has to use appropriate analysis techniques and methodologies that allows access to this region in order to probe and extract useful information. This is rather challenging, as conventional hybrid systems are composed of a thick (*μ*m) polymer layer on one side coupled with a thick metal substrate on the other side. The resulting structure is a buried interface, which is difficult to characterize with surface-sensitive analysis techniques. It was shown that by careful fitting of high-resolution XPS spectra, the XPS analysis technique can be used to probe acid-base interactions of polymers on top of a metal oxide substrate by depositing the polymer as an ultrathin film^12–21^. Moreover, many common surface-sensitive analysis techniques require the detector to operate under vacuum conditions, making it challenging to probe environmental effects *in situ*.

The work shown here is part of ongoing research, where the initial effects of water on interfacial interactions of different acrylic polymers are investigated. Previously, we reported how interfacial interactions on the acrylic polymer and hydroxylated aluminium oxide interface were probed using ambient pressure X-ray photoelectron spectroscopy (APXPS). Our study confirms the formation of carboxylate anion bonds by deprotonation, as earlier reported for carboxylic acids^22–28^. Moreover, for the first time, it was reported that the amount of bonds increased when water was present. Therefore, APXPS has been validated as an *in situ* probe for the effect of water on the interfacial chemistry using carboxylic polymers. In this work an additional step is taken, as the adsorption of a more complex polymer structure on a metal oxide surface, poly(methyl methacrylate) (PMMA), is studied. PMMA has an ester functional group, more specifically a methyl methacrylate, which leads to a large difference in macroscopic properties with respect to PAA, such as a high resistance to water.

One of the first investigations of the polymer/metal oxide interface with XPS was performed by Chemini and Watts^29^. They investigated PMMA films that were peeled from silicon and glass substrates, showing that a reorientation of the carboxyl group of PMMA exists at the interface. Leadley and Watts investigated ultrathin PMMA films on several native metal oxides showing that PMMA interactions with metal oxide substrates are determined by the acido-basic nature of the substrate^30,31^.

The interaction of PMMA with different metal oxide substrates has been investigated by simulation and experimental methodologies^22,32–37^. However, different interfacial interaction mechanisms were suggested and various methodologies to reach the interface were utilized. It was also stated that one of the most important aspects of the adhesion process of PMMA/aluminum oxide remains unanswered. This aspect is to what extent the formed chemical bonds at the interface are irreversible as an inevitable consequence of hydrolysis and how strongly the chain segments remain in place. The role of water as a mediating agent in the adsorption process was theoretically suggested but not elucidated experimentally^29,30,36–39^.

The object of this work is to characterize the formed interfacial bonds, investigate the influence water has on these acid-base interactions and to propose a reaction mechanism of the changes water molecules induce on the interfacial chemistry. In the discussion of this work, the observations made for the PMMA/aluminum oxide system are compared with the observations made previously on the PAA/aluminum oxide system to highlight the different surface interactions of ester and carboxylic acid functional groups. Due to a difference in acid-base interactions, different types of interfacial bonds are expected. Here, these interfacial interactions are identified and their different behaviour in the presence of water is investigated.

A new approach to probe chemical interactions of buried polymer/metal oxide interfaces is made possible due to an ongoing development of the APXPS technique^40,41^. Thanks to differentially pumped lensing stages, a wide range of water vapour pressures (or relative humidities (RH)) can be reached in the analysis chamber, permitting the investigation of interfacial chemistry changes of organic/inorganic systems *in situ* ^2^. In the APXPS chamber the maximum humidity exposure for this system is limited to 5 Torr due to strong attenuation of the signal by the gas phase in the analysis chamber. ATR-FTIR in the Kretschmann geometry allows an interface-sensitive and *in situ* analysis of a polymer/metal oxide interface^42–44^. Additional information of the molecular structure at the interface of the thin polymer film coatings is obtained by ToF-SIMS measurements.

For the APXPS and ToF-SIMS experiments, a model coating was applied on a metal oxide surface formed on a rolled metal sheet. Whereas for the ATR-FTIR Kretschmann experiments, the coating is applied on an evaporation deposited model metal oxide, which is then exposed to an excess of water. These model systems have a different metallurgy and the humidity levels that can be reached in the analysis chambers of both techniques are different. However, these techniques are complimentary. Their combination allows for characterization of the formed bonds at the interface, demonstration of the effect of water at the interface *in situ*, and the proposal of a bonding mechanism. The main advantage of the presented investigation is that the selection of these different model approaches allow us to only probe chemical changes in interfacial interactions that are induced by humidity. Other processes (e.g. cathodic or stress-induced delamination) that also might occur simultaneously during macroscopic adhesion tests under humid conditions are therefore not interfering.

By combining the observations made with these different (*in situ*) spectroscopic techniques and by comparing the obtained results with the study of the carboxylic acid model system (Pletincx *et al*.^2^), a complete adsorption mechanism is proposed. This mechanism shows the nature of the different formed bonds between the ester functional group (methyl methacrylate) of the polymer and the surface hydroxyl groups of aluminum oxide when water is present at the interface. The comparison of polymers with different functional groups allow us to understand what local chemical interactions govern good adhesion under humid conditions and thus on a macroscopic level lead to a prolonged coating durability under realistic, day-to-day environments.

## Experimental Methods

### Materials and sample pretreatment

0.3 mm thick ultrapure aluminum substrates (99.99% metals basis, rolled sheet, Norsk Hydro Aluminium) are used for the APXPS and ToF-SIMS study. Samples were rinsed ultrasonically in acetone for 5 min. Next, the samples were chemically etched in a 25 g L^−1^ NaOH solution at 70 °C for 1 min. Then the samples were rinsed with water, blown dry with nitrogen, and exposed to atmosphere in order to form a native oxide layer. PMMA ultrathin films were prepared by exposure of the aluminum substrates in a 0.1% w/w PMMA (Mw = 250.000 g mol^−1^) toluene solution for 24 h. After the deposition process, the samples were removed from the solution and placed in pure solvent to remove physisorbed polymer chains. This leads to an oxide surface covered with chemisorbed polymer chains. For the ATR-FTIR in Kretschmann geometry measurements, a germanium internal reflection element (IRE) hemisphere was coated with a 50 nm thick pure aluminum layer (99.99% metals basis, pellets, Johnson Matthey) by means of a high-vacuum evaporation coating system (Balzers BAE 250). The film thickness was determined with a quartz crystal microbalance. After deposition, the metal film was exposed to ambient conditions to form a passivated oxide layer. The polymer adsorption process was followed *in situ* at the interface by bringing the PMMA/toluene solution in contact with the metal coated IRE crystal. For the water ingress measurements, the metal coated IRE crystal was first coated with the polymer by drop casting of the 0.1% w/w PMMA/toluene solution, followed by a solvent rinse. This PMMA/aluminum oxide coated IRE crystal was then exposed to a 0.1 M borate buffer to investigate the effect of water at the interface.

### Interface Characterization

#### Ambient pressure X-ray photoelectron spectroscopy (APXPS)

The spectra of the ultrathin PMMA on aluminum oxide substrates were acquired on an ambient pressure X-ray photoelectron spectrometer (SPECS Phoibos 150 NAP). Time between the deposition of the PMMA films and the introduction of the samples in the analysis chamber was kept as small as possible. At room temperature, ambient gas (oxygen, nitrogen, carbon dioxide, …) is dissolved in liquid water. Since it is desired to only dose water molecules in the analysis chamber, water (18.2 M Ω cm) was degassed by three cycles of liquid nitrogen freeze-pump-thaw before introduction into the APXPS analysis chamber from a glass vessel via a precision leak valve. A pass energy of 20 eV and an energy step of 0.05 eV were used to record the spectra of the different photoelectron core levels. The X-ray source (Al K-alpha, monochromatized, SPECS MF 60) was set at an acceleration voltage of 14 kV and a power of 100 W was used for the irradiation of the organic/inorganic system. The fitting procedure was performed by utilizing KolXPD software. Peak shapes consisted of a Voigt mixture of Gaussian/Lorentzian, with a Shirley type background. The binding energy scale was calibrated by setting the aliphatic (*C−C/C−H*) component of the C 1 s peak to 284.8 eV after fitting.

#### Fourier transform infrared spectroscopy in the Kretschmann geometry (ATR-FTIR Kretschmann)

Infrared spectra were recorded with a Thermo-Nicolet Nexus Fourier transform infrared spectroscopy (FTIR) apparatus equipped with a mercury-cadmium-telluride (MCT) liquid-nitrogen-cooled detector, and a nitrogen-purged measurement chamber with a Harrick Seagull multipurpose reflection accessory. The resolution of the acquired spectra is 8 cm^−1^. The control of the spectra acquisition was managed by the OMNIC 8.1 software package (ThermoElectron Corporation, Madison, WI). A spectrum of the IRE crystal coated with aluminum oxide against pure toluene was taken as a background prior to the polymer deposition and polymer solution exposure. For the water ingress measurements, a spectrum of the IRE crystal coated with aluminum oxide against ambient conditions was taken as a background prior to polymer deposition and prior to electrolyte exposure.

#### Time-of-Flight Mass Spectrometry

ToF-SIMS measurements were performed using a TOF.SIMS 5 (ION-TOF GmbH). Static SIMS conditions with a total ion dose less than 1 × 10^13^ *ions cm* ^−2^ *analysis* ^−1^ were employed using a 30 kV $B{i}_{3}^{+}$ primary ion beam operating in the high current bunched mode for high spectral resolution. An analysis area of 100 × 100 *μm* ^2^ at a resolution of 128 × 128 pixels was used. ToF-SIMS spectra were acquired over a mass range of 1–850*u* in both positive and negative ion modes. Fragments of known composition, such as $C{H}_{3}^{+}$, ${C}_{2}{H}_{2}^{+}$, $AlO{H}^{+}$, ${C}^{-}$, $C{H}_{2}^{+}$, ${C}_{2}{H}_{3}{O}^{-}$ and $Al{O}^{-}$ were used for mass calibration and carefully checked to confirm that these peak shapes did not show any charging effect.

## Results and Discussion

### Characterization of the ultrathin PMMA/aluminum oxide interface

#### Monitoring adsorption of PMMA at the aluminum oxide interface from a polymer solution *in situ*

The buried hybrid interface was monitored by *in situ* ATR-FTIR during the exposure of the oxidised aluminum coated IRE crystal to a PMMA/toluene solution. By conducting this experiment, an interface spectrum is obtained of the interface between the adsorbed polymer and the hydroxide surface, while the deposition process is occurring. The oxide-coated IRE crystal in contact with pure toluene (no PMMA in solution) is used as the background spectrum.

**Figure 1:**
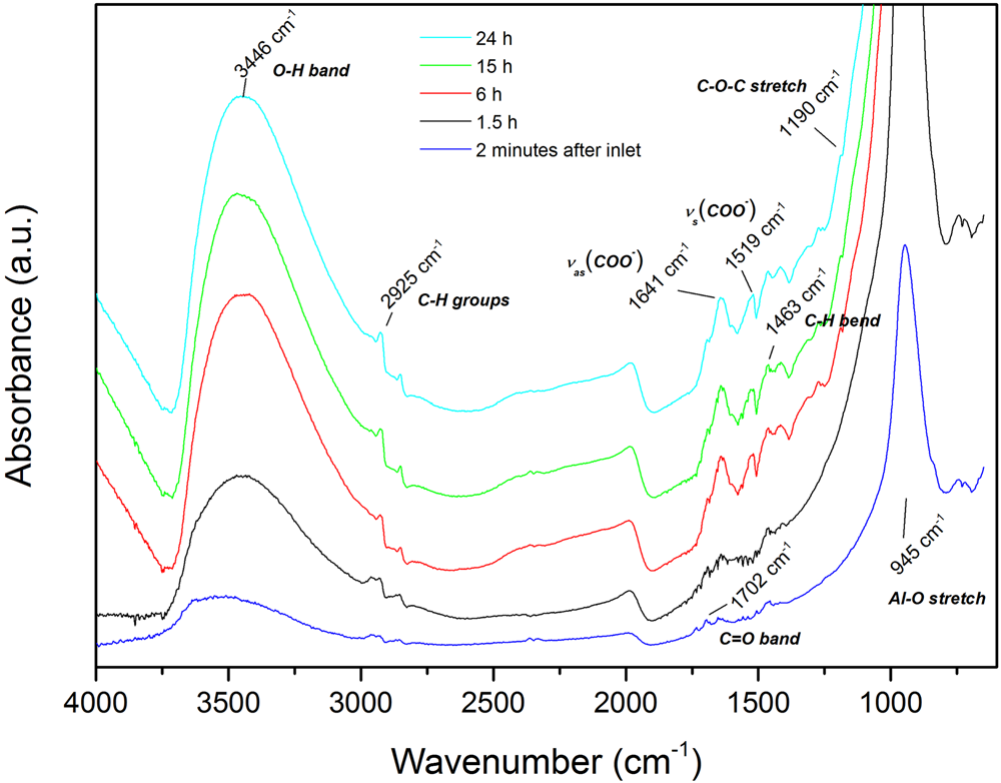
ATR-FTIR Kretschmann spectra of a PMMA/toluene solution on native aluminum oxide. Spectra were taken at different solution exposure times to follow the adsorption of the polymer on the aluminum oxide surface *in situ*.

The interface spectra are presented in Fig. 1 with increasing adsorption time during the first 24 h after the inlet of the polymer solution. The spectra show a peak at 3446 $c{m}^{-1}$ that increases in intensity with increasing exposure time. This peak corresponds to the appearance of $OH$ containing species at the interface. The peak observed at 945 $c{m}^{-1}$ is from the $Al-O$ and $Al-OH$ bending vibrations^9,45,46^. It has been shown previously by van den Brand *et al*. that the aluminum oxide surface continues ageing in the first 20 hours after deposition due to adsorbed water from the ambient and causes growth and hydroxylation of the oxide layer. It was also stated that this water film can not be removed by solvents^47^. This corresponds to the increase over time of the $Al-O$ and $Al-OH$ peaks. The increasing $OH$ peak (at 3446 $c{m}^{-1}$) is consistent with the increase of the $Al-O$ and $Al-OH$ peak. Peaks around 2925 $c{m}^{-1}$ are also observed, which correspond to $C{H}_{2}$ stretching vibrations, together with the $CH$ bending at 1463 $c{m}^{-1}$ showing the presence of the polymer at the oxide/solution interface. The carbonyl vibration band ($C=O$) is observed at 1702 $c{m}^{-1}$, which is at a lower wavenumber than observed in the FTIR spectra of bulk PMMA. For bulk PMMA, the carbonyl group is observed at 1732 $c{m}^{-1}$ ^48^. The observation at lower wavenumber (1702 $c{m}^{-1}$) is a result of hydrogen bonding of the carbonyl group at the aluminum oxide surface^9,49^. This shows that there is a certain orientation of these groups at the interface, which also results in the presence of a small $C-O-C$ stretching vibration at the interface at 1190 $c{m}^{-1}$ ^48^. The interface sensitivity of the ATR-FTIR technique in Kretschmann configuration is emphasized here, since it is known that the bulk spectra of PMMA contain large carbonyl (1732 $c{m}^{-1}$) and ether bands (1190 $c{m}^{-1}$), whereas in these spectra the peaks have a low intensity with respect to other peaks^48^. The bands occurring in the range around 1450–1650 $c{m}^{-1}$ indicate that a carboxylate structure is coordinatively bonded to the aluminum oxide^36,39,50–52^. The peak at 1641 $c{m}^{-1}$ is assigned to the ${\nu }_{as}(CO{O}^{-})$ carboxylate stretch. The symmetric carboxylate stretch ${\nu }_{s}(CO{O}^{-})$ can be found at 1519 $c{m}^{-1}$. Methanol formation at the interface during the adsorption reaction is a mechanism that has been proposed previously^36,39^. We also observe evidence of methanol formation in this study, through observation of carboxylate peaks and the relative smaller $C-O-C$ peak. Because of the observation of the carboxylate peaks, it can be stated that part of the observed $OH$ peak also results from methanol formed at the interface due to the adsorption reaction occurring at the interface. Since the $C-O-C$ peak at the interface is small with respect to the carboxylate peaks, this is a second indication that the methoxy group has reacted at the interface and was split off to form methanol. More evidence for the split-off of the methoxy group and thus the formation of methanol was observed by ToF-SIMS (shown in section 3.1.3).

Thus, the *in situ* adsorption spectra show that hydrogen bonds and carboxylate ionic bonds are formed and suggest that methanol has been split off at the PMMA/aluminum oxide interface over the course of 24 h. The formation of a chemically-bound PMMA coating by the reactive adsorption deposition method was achieved.

#### Characterization of the interfacial interactions between ultrathin PMMA/aluminum oxide by APXPS

**Figure 2:**
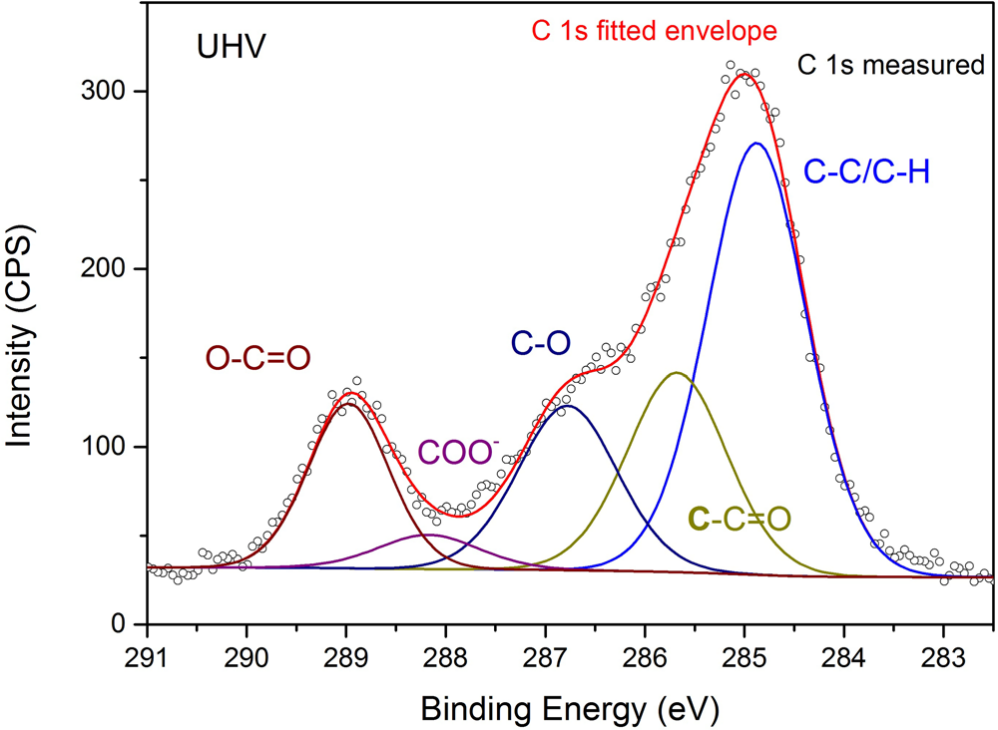
C 1 s XPS spectra of an ultrathin PMMA film on native aluminum oxide at UHV. The spectrum is fit with components assigned to $C-C/C-H$, $C-C=O$, $C-O$, $CO{O}^{-}$ and $O-C=O$ contributions. The $CO{O}^{-}$ peak corresponds to the ionic bond formed at the polymer/metal oxide interface.

The C 1 s XPS spectrum of bulk PMMA is typically fit with four peaks^53^. These four peaks are indeed required to fit our spectra, as shown in Fig. 2, with following binding energies: $C-C/C-H$ (284.8 eV), an beta carbon peak $C-C=O$ (+0.8 eV BE shift with respect to $C-C/C-H$), $C-O$ (+1.9 eV BE) and $O-C=O$ (+4.1 eV BE). However, this four-peak strategy is not sufficient to fit our spectra. One additional peak is required to fit the spectra as was done by Leadley *et al*.^30^. This peak corresponds to the carboxylate species ($CO{O}^{-}$), which results from the formation of ionic bonds at the hybrid interface, and has a BE shift of +3.3 eV relative to the aliphatic carbon peak. This BE shift is in accordance with literature values (+3.2 and +3.5 eV) obtained from thin acrylic polymers adsorbed on metal oxides^20,21,25,30,31^. The FTIR results described in the previous section, where the carboxylate anion symmetric and asymmetric peaks are observed and ToF-SIMS data (shown in section 3.1.3) provide additional justification for the use of a carboxylate peak when fitting the C 1 s XPS spectra.

The intensity of the beta carbon peak ($C-C=O$) was constrained during the fitting procedure to equal the sum of the carboxylate ($CO{O}^{-}$) and carboxyl carbon ($O-C=O$) components. This constraint was done since every $O-C=O$ and $CO{O}^{-}$ group has one adjacent beta-carbon based on the structure of the polymer. It is observed that the intensity of the $C-C/C-H$ peak is slightly larger than would be expected for PMMA based on the intensity of the $O-C=O$ peak, whereas the expected ratio from the PMMA molecular structure is 2:1. In literature, this increased $C-C/C-H$ intensity was explained to be an excess of hydrocarbons from the polymer itself or to adventitious hydrocarbon^20,21^. However, in these cited works the researchers were unable to differentiate the origin of this increase. It is known that surface carbon contamination is typically replaced at the interface during the polymer deposition process, we attribute this extra intensity to aliphatic adventitious carbon on top of the newly formed polymer film^54^. We assume that the effects of adventitious carbon on the interfacial chemistry is small since this is a small contribution to the surface species and is not primarily located at the polymer/oxide interface, as we assumed in our previous investigation^2^. In our observations, we see a binding energy shift of the $O-C=O$ peak of +4.1 eV whereas for bulk PMMA, this peak shift is +4.0 eV. This BE shift indicates that the unbound functional groups of the PMMA, more specifically the carbonyl oxygen, form hydrogen bonds with the aluminum oxide surface. This observation is in agreement with the observations from the ATR-FTIR Kretschmann PMMA adsorption experiments, where the $C=O$ peak was observed at lower wavenumbers. It was shown by Beamson *et al*. that the carboxyl carbon in ultrathin films deposited on silicon has a binding energy shift of 0.1 eV greater than that observed in bulk PMMA^55^. They have shown, by additional infrared spectroscopy experiments, that this increase in binding energy shift is due to hydrogen bonding between the carbonyl oxygen of PMMA and silanol groups of the substrate^49^. Here, it can thus be concluded that a combination of hydrogen bonds and ionic bonds are formed at the PMMA/aluminum oxide interface.

#### ToF-SIMS measurements to obtain molecular structure information of ultrathin PMMA/aluminum oxide interface

**Figure 3:**
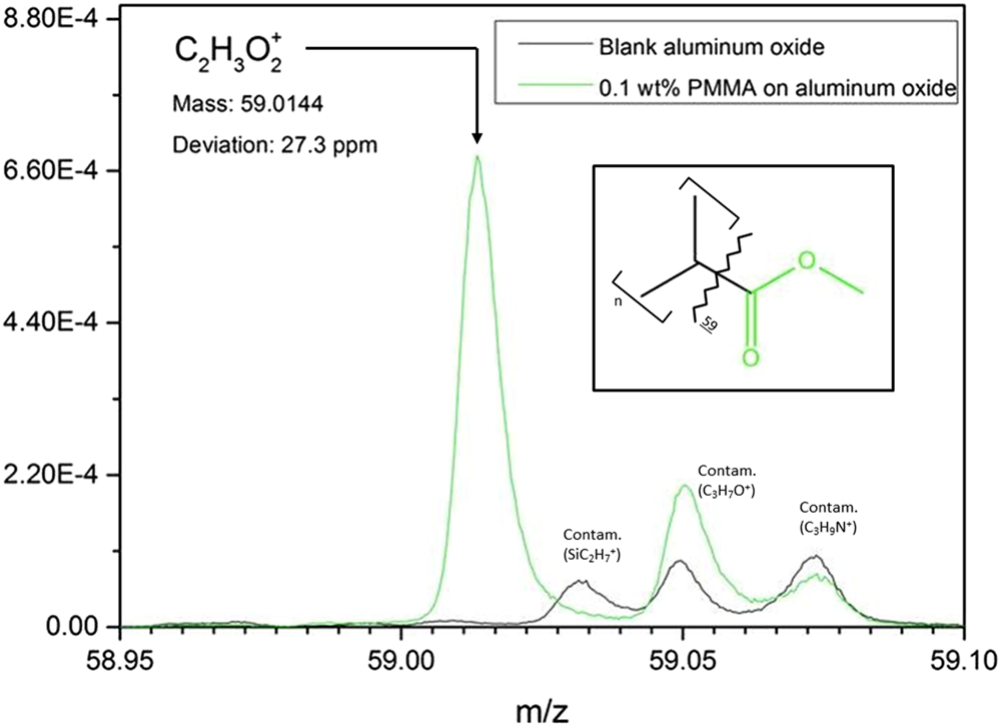
ToF-SIMS spectra showing a characteristic peak (${C}_{2}{H}_{3}{O}_{2}^{+}$) for PMMA. This observation shows that the polymer was successfully deposited from the polymer solution and that PMMA can be distinguished from carbonaceous contamination on the aluminum oxide surface. Inset: schematic representation of fragmentation of the monomer unit with the observed ion fragment coloured in green. Mass is the experimentally obtained mass. The deviation (ppm) is the difference between the experimental and the theoretical mass.

Mass spectra were obtained from an ultrathin PMMA overlayer on aluminum oxide and from an uncoated aluminum oxide sample. The blank is analysed to obtain reference spectra. Figure 3 shows a selection of the acquired positive mass spectra. A fragment characteristic for the coated sample is obtained at $m/z\mathrm{=59}u$ and can be assigned to ${C}_{2}{H}_{3}{O}_{2}^{+}$ with a mass accuracy of 27.3 ppm. ${C}_{2}{H}_{3}{O}_{2}^{+}$ is likely formed by fragmentation of PMMA functional groups, as shown in the inset of Fig. 3. The presence of this peak proves that the deposition method was successful and that PMMA can be distinguished from carbonaceous contamination on the aluminum oxide surface by ToF-SIMS. We assign this fragment (${C}_{2}{H}_{3}{O}_{2}^{+}$) to the methoxy and carbonyl functional group of PMMA.

**Figure 4:**
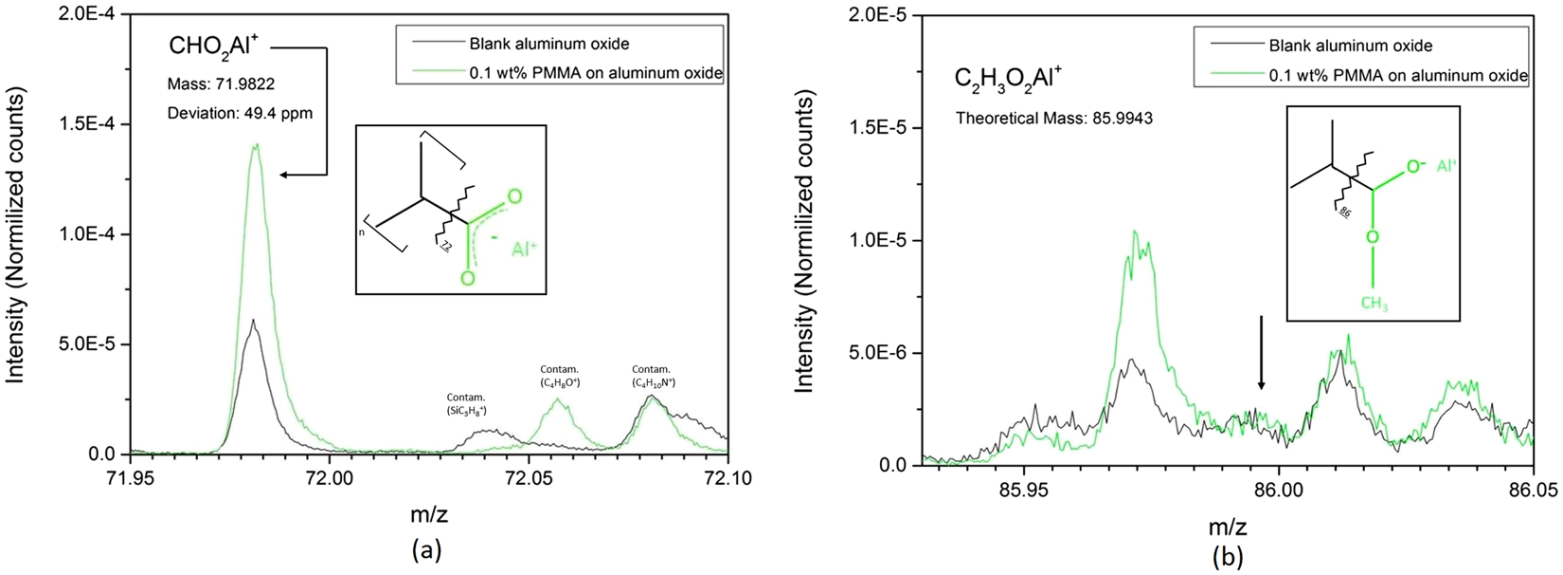
(**a**) ToF-SIMS spectra showing a fragment ($CH{O}_{2}A{l}^{+}$) that indicates the carboxylate bond between PMMA and aluminum oxide showing that the polymer is ionically bonded to the metal oxide. (**b**) ToF-SIMS spectra showing the absence of a theoretical fragment (${C}_{2}{H}_{3}{O}_{2}A{l}^{+}$), that would be expected if the methoxy group of PMMA was present when the functional group is bonded to the interface. Insets: schematic representations of fragmentation of the monomer unit with ion fragment coloured in green. Mass is the experimentally obtained mass. The deviation (ppm) is the difference between the experimental and the theoretical mass.

Because the PMMA film is made sufficiently thin, substrate and interface fragments are also acquired. Therefore, identifying fragments that originate from the interfacial bond is possible. In Fig. 4a, a fragment at $m/z=72u$ is shown. This fragment is assigned to $CH{O}_{2}A{l}^{+}$ and can be related to the carboxylate anion bond. These observations are in accordance with the observations provided by APXPS and ATR-FTIR Kretschmann, where we also observed the formation of this type of bond.

From the ATR-FTIR Kretschmann measurements, it was already indicated that the methoxy group splits off at the interface as a consequence of carboxylate bond formation, resulting in methanol being formed. In order to sustain this theory, we scanned the mass spectra to find a fragment that still contained the methoxy group while this group was bonded to the aluminum oxide. No such fragment was found in the positive mass spectra and neither in the negative mass spectra (example shown in Fig. 4b). Absence of such fragments is in accordance with the ATR-FTIR spectra, where a rather small $C-O-C$ stretching vibrations was observed and where the $OH$ peak thus partly originates from the formed methanol at the interface. The ToF-SIMS measurements in combination with the ATR-FTIR Kretschmann measurements validate the used model for the APXPS fitting since these results show the presence of the carboxylate anion. Thus, validating the use of the ionic bond’s corresponding peak in the model of the C 1 s APXPS spectra.

### The effect of water on the interface of PMMA/aluminum oxide

#### The PMMA/aluminum oxide interface exposed to an aqueous electrolyte, monitored by *in situ* ATR-FTIR Kretschmann

The uptake of an electrolyte through the polymer coating and the resulting chemical changes at the buried interface are probed *in situ* by ATR-FTIR in the Kretschmann geometry.

**Figure 5:**
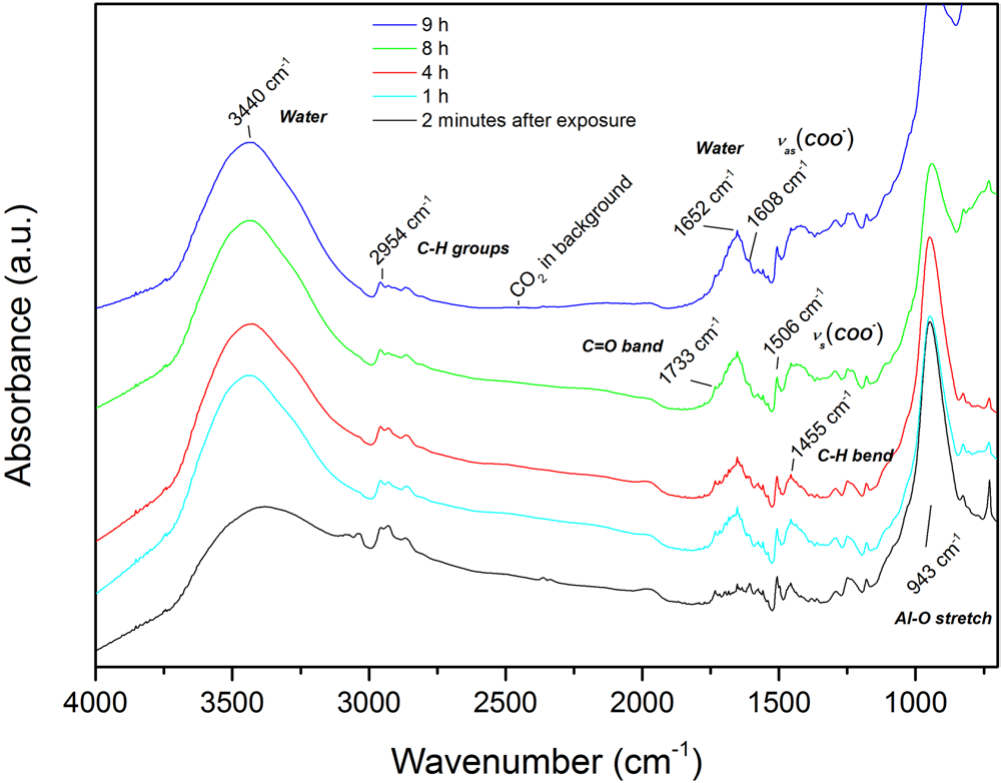
ATR-FTIR Kretschmann spectra of PMMA on native aluminum oxide. Spectra were taken at different times before and after exposure to a 0.1 M borate buffer solution during the first 9 h.

In Fig. 5, the IR spectra are shown of a 0.1 wt% PMMA adsorbed on an aluminum oxide coated germanium crystal when a 0.1 M borate buffer was used as an electrolyte. The IR background was that of an aluminum oxide coated germanium crystal, prior to polymer deposition in contact with ambient air.

Analysing the spectrum just after electrolyte has been introduced to the system (Fig. 5) the peaks present around 1450–1650 $c{m}^{-1}$ indicate that carboxylate bonds were formed at the interface^36,39,50–52^. The peak at 1608 $c{m}^{-1}$ is assigned to the ${\nu }_{as}(CO{O}^{-}$) carboxylate stretch. The symmetric carboxylate stretch ${\nu }_{s}(CO{O}^{-})$ can be found at 1506 $c{m}^{-1}$. The peak at 956 $c{m}^{-1}$ is assigned to the $\nu (Al-O)$ of free surface hydroxyl groups on aluminum oxide^9,45,46^. The bands between 3000–2800 $c{m}^{-1}$, together with the peak 1455 $c{m}^{-1}$ are assigned to $C{H}_{2}$ stretching, indicating the presence of $C{H}_{2}$ of the polymer chain at the interface.

In Fig. 5, the interface spectra are also shown at different electrolyte-exposure times up to 9 h. The first observation that can be made is the immediate increase of peaks located at 3440 and 1652 $c{m}^{-1}$. These peaks are characteristic for water, which accumulates at the interface over time. The symmetric and asymmetric stretch of the carboxylate are present and also increase in time. Simultaneously, the $Al-O/Al-OH$ peak located at 956 $c{m}^{-1}$ decreases in time, when water is diffusing towards and reacting at the interface. These observations show that water enables/enhances the formation of carboxylate anions at the interface, while free surface $OH$ groups are being consumed. The $C=O$ peak is immediately observed at a higher wavenumber (1733 $c{m}^{-1}$) than what was the case for the adsorption experiments (1702 $c{m}^{-1}$). The carbonyl band is observed at the wavenumber for bulk PMMA, showing that the formed hydrogen bonds located at the interface are replaced. Water molecules thus immediately replace the hydrogen bonds formed at the interface and mediate the formation of more carboyxlate ionic bonds.

#### Ultrathin PMMA depositions on aluminum oxide exposed to varying relative humidities, investigated by APXPS

**Figure 6:**
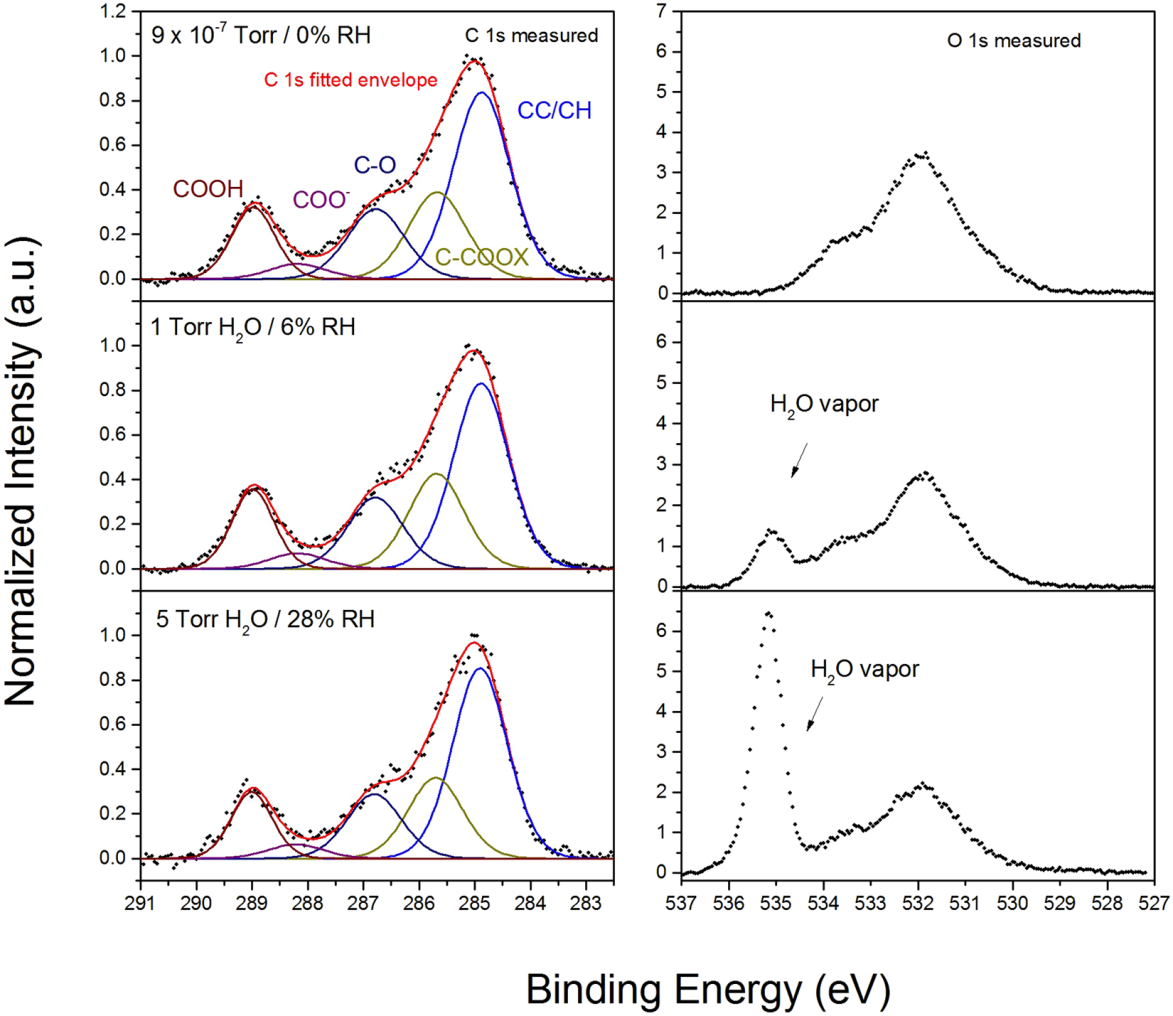
Normalized C 1 s (left) and O 1 s (right) APXPS spectra of an ultrathin PMMA film on native aluminum oxide at varying water vapor pressures. 9 × 10^−7^ Torr (**a**), 1 Torr H_2_O (**b**), 5 Torr H_2_O (**c**).

Different water vapour pressures were attained in the APXPS analysis chamber in order to simulate the effect of water on the PMMA/aluminum oxide samples. Three analysis conditions were achieved by dosing different amounts of water to the analysis chamber, being ultra high vacuum, a low relative humidity (6% RH), and a higher relative humidity (28% RH). Above 5 Torr (28% RH) vapour pressure, the attenuation of the photoelectron signal is very strong, leading to a very poor signal-to-background ratio. A relative humidity of 28% in the analysis chamber is thus the maximum value that can be achieved with this APXPS setup. In Fig. 6, the normalized C 1 s and O 1 s peak intensities for surface species are shown as the water vapour pressure is increased. The C 1 s spectra is fit with peak components assigned to $C-C/C-H$, $C-C=O$, $C-O$, $CO{O}^{-}$ and $O-C=O$ species, as described in section *section 3*.*1*.*2*. In the O 1 s spectra, the water vapour phase peak is observed at higher BE than the contributions from the surface species. A broad band containing contributions from aluminum (hydr)oxide and PMMA. After exposure to water vapour, surface hydroxyls and/or adsorbed water contributions are also observed. Deconvolution of this broad peak is challenging since the many individual contributions forming the O 1 s peak are not well resolved. However, no significant changes, except for the change in the water vapour peak, are observed. Therefore, we are limited to the interpretation of the C 1 s spectra.

For the deposited PMMA films on metal oxides, one additional C 1 s peaks is required during fitting in comparison with the spectrum of the bulk polymer as was discussed in *section 3*.*1*.*2*. When water vapour is introduced in the analysis chamber, the relative intensities of the fitted C 1 s peaks show little changes and no BE shifts occur during water exposure. The relative intensity of the carboxylate peak does not increase. When calculating the degree of conversion of carboxylic acid to carboxylate, a value of 0.2 is obtained at 9 × 10^−7^ Torr, 1 Torr and 5 Torr. This degree of conversion is calculated by dividing the area of the carboxylate peak by the sum of the carboxylate and carboxylic acid peak ($[CO{O}^{-}]/([CO{O}^{-}]+[O-C=O]$). When we studied PAA on aluminum oxide substrates, we observed a change in relative intensities of the carboxylate and carboxylic acid peaks. Also a degree of conversion of 0.5 was observed, which is about 50% lower than that of PMMA. These observations can be explained as that PMMA needs more water to bond to the aluminum oxide. First a hydrolysis reaction has to split off the methoxy group and form methanol (as was shown with ATR-FTIR and ToF-SIMS), hereafter another water molecule has to deprotonate the carboxylic acid functional group to form the carboxylate anion. This second step was also observed when investigating PAA/aluminum oxide systems^2^.

### Schematic representation of PMMA adsorption on aluminum oxide and the effect of water molecules on chemical interactions at the hybrid interface

**Figure 7:**
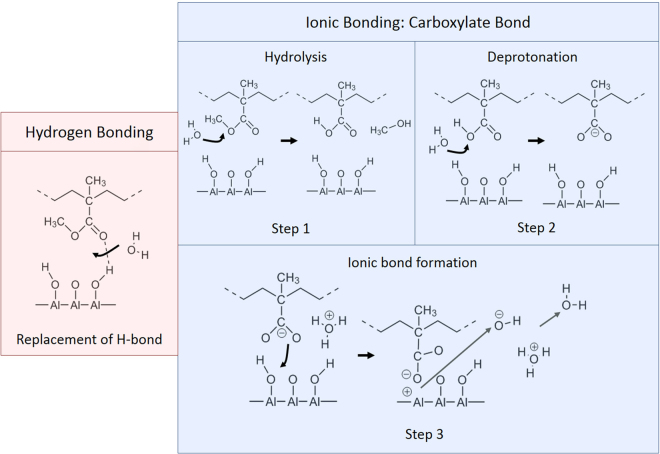
Schematic representation of the adsorption mechanism of PMMA on native aluminum oxyhydroxide and the effect of water on the formed bonds. Hydrogen bonds between the carbonyl group of PMMA and the surface hydroxyl groups are replaced by water. The carboxylate ionic bond formation is enabled/enhanced by water molecules at the interface. First a hydrolysis of the methoxy group takes place forming methanol (Step 1). Then the carboxylic acid group is deprotonated to form a carboxylate anion (Step 2), which reacts with a free surface hydroxyl group resulting in an ionic bond at the interface (Step 3). In this schematic representation, the coordination type of the ionic bond is not taken into account.

The adsorption process of PMMA on the aluminum oxide surface and the effect of water on the different chemical interactions at the interface are discussed here. This is done based on the observations made with *in situ* ATR-FTIR Kretschmann (from the adsorption experiments and the electrolyte influence), *in situ* APXPS experiments at different water vapour pressures, and ToF-SIMS observations at high vacuum conditions. In this work it is shown that APXPS can be used to identify hydrogen bonds and ionic bonds at the polymer/metal oxide interface, but no changes are observed when the water pressure in the analysis chamber is increased up to a RH of 28% (the pressure limit of this APXPS setup). Whereas for PAA, it was shown that the amount of carboxylate anions increases up till a pressure of 5 Torr^2^. Complementary to this technique, *in situ* ATR-FTIR in the Kretschmann geometry is utilized to characterize the formed bonds during reactive adsorption and to describe the effect of water on the interface. This technique shows that the amount of carboxylate ionic bonds at the PMMA/aluminum oxide, just as was observed for the PAA/aluminum oxide interface, increases when an excess amount of water was introduced. These observations show that more water is required for PMMA to bond to aluminum oxide than it is for PAA to bond. This is explained by the need of water to first hydrolyse the methoxy group present for PMMA to form a carboxylic functional group and methanol. The implification of methanol formation was shown in this work by ATR-FTIR Kretschmann and ToF-SIMS. After the hydrolysis step, the formed carboxylic acid functional group is deprotonated, forming a carboxylate anion group that bonds with the aluminum oxide substrate. For PAA, only a deprotonating step is required to bond to the aluminum oxide surface. Theoretically this means that PMMA needs twice the amount of water molecules at the interface to bond to the substrate than PAA. APXPS and ATR-FTIR Kretschmann measurements also showed that the carbonyl group of the non-ionically bound functional groups form hydrogen bonds with the aluminum oxide surface (shown in Fig. 7), but it was also shown that when water molecules diffuse to the interface it immediately replaces the formed hydrogen bonds.

ToF-SIMS mass spectra provided an additional proof of the carboxylate anion bond and indicate hydrolysis of the ester bond (and thus methanol formation) during adsorption of PMMA. A schematic representation of the adsorption mechanism is shown in Fig. 7. The observations show that water plays an important role at the interface. A water molecule first hydrolyses the methoxy group to form methanol at the interface. Then another water molecule deprotonates the formed carboxylic acid functional groups creating hydroxonium ions. The formed carboxylate species react with the surface hydroxyl groups. More water at the interface leads to more ionic bond formation. At the same time water replaces the hydrogen bonds formed with the carbonyl group of the non-ionically bound functional groups. This proposed adsorption mechanism based on our *in situ* measurements is in agreement with the *ex situ* observations and suggestions made by Konstandinidis *et al*., Tannenbaum *et al*. and Leadley *et al*.^25,36,39^.

## Conclusion

Bond formation and the effect of water on the different interfacial interactions of a PMMA/aluminum oxide interface are elucidated experimentally. Here, we showed that ATR-FTIR Kretschmann, ToF-SIMS and APXPS can be used to identify chemical interactions locally at the hybrid interface. *In situ* ATR-FTIR in the Kretschmann geometry was utilized to characterize the formed hydrogen and ionic bonds during reactive adsorption and to describe the effect of water on the interface. This technique shows that the amount of carboxylate ionic bonds increases when water was introduced and that free surface hydroxyl groups are consumed in the process. When water reaches the interface, the water molecules immediately replace the formed hydrogen bonds between the carbonyl group and the surface hydroxyl groups of aluminum oxide. No changes were observed at high water vapour pressures with APXPS up to 5 Torr (a RH of 28%). The observations made with these techniques allow us to schematically represent the adsorption process of PMMA when water is present at the interface. Water plays a pivotal role at the interface, where hydrolysis of the methoxy group leads to the formation of methanol and a carboxylic acid functional group. The carboxylic acid functional groups are then deprotonated to form carboxylate ionic bonds. At the same time water replaces the formed hydrogen bonds immediately at the interface.
